# Association between Asymptomatic Unilateral Internal Carotid Artery Stenosis and Electrophysiological Function of the Retina and Optic Nerve

**DOI:** 10.1155/2017/4089262

**Published:** 2017-04-11

**Authors:** Anna Machalińska, Aleksandra Kowalska-Budek, Miłosz Piotr Kawa, Arkadiusz Kazimierczak, Krzysztof Safranow, Marta Kirkiewicz, Grażyna Wilk, Wojciech Lubiński, Piotr Gutowski, Bogusław Machaliński

**Affiliations:** ^1^Department of Ophthalmology, Pomeranian Medical University, Szczecin, Poland; ^2^Department of General Pathology, Pomeranian Medical University, Szczecin, Poland; ^3^Department of Vascular Surgery and Angiology, Pomeranian Medical University, Szczecin, Poland; ^4^Department of Biochemistry and Medical Chemistry, Pomeranian Medical University, Szczecin, Poland; ^5^Department of General and Dental Radiology, Pomeranian Medical University, Szczecin, Poland

## Abstract

*Purpose*. This study was designed to assess retinal and optic nerve bioelectrical function in patients with unilateral asymptomatic but hemodynamically significant internal carotid artery stenosis (ICAS). *Methods*. Forty-two subjects with a diagnosis of unilateral ICAS and 34 controls were analyzed. Full-field electroretinogram (ERG), pattern electroretinogram (PERG), and pattern visual-evoked potentials, as well as optical coherence tomography and ophthalmological examination, were performed. Data analysis included eyes ipsilateral to ICAS (EIS) and eyes contralateral to ICAS (ECS). *Results*. Intraocular pressure was significantly decreased in EIS and ECS compared to that in the controls. In the macula, both the cube average thickness and cube volume values were significantly reduced both in EIS and ECS compared to those in the controls. Similarly, PERG P50 and N95 wave amplitudes were significantly smaller in EIS and ECS compared to those in the controls. The ERG rod b-wave and rod-cone a-wave amplitudes were decreased, and implicit times were significantly prolonged, whereas the OP wave index was reduced in EIS compared to that in the controls. No differences in IOP, OCT, or ERG and PERG parameters were identified between EIS and ECS. *Conclusions.* Our study demonstrated that retinal bioelectrical function is negatively affected by ICAS despite the absence of objective clinical signs and symptoms of ocular ischemia.

## 1. Introduction

The management of patients with asymptomatic carotid artery stenosis is one of the most controversial problems in clinical practice. The outcomes of the Asymptomatic Carotid Atherosclerosis Study (ACAS) have indicated that carotid endarterectomy (CEA) reduces the risk of stroke compared with noninvasive management [1]; however, the potential risks of angiography and vascular surgical intervention may not justify their introduction into routine clinical strategies. The benefit of the CEA procedure may be meaningfully increased in patients with a high predisposition to develop cerebrovascular events [2]. To date, defining a high-risk patient with asymptomatic severe ICAS remains problematic, and it is necessary to establish reliable tests that may be used to identify hemodynamically impaired patients, particularly in the subclinical phase of ischemia.

The retina is a light-sensitive tissue of the central nervous system and is composed of several types of cells. The functional signals and activities of each cell type may be recorded via electroretinography (ERG). ERG is a sensitive tool used to detect ischemic injury, and it is well established how retinal ischemia may affect different retinal cells and their functions. The b-wave of flash ERG is a particularly sensitive index of retinal ischemia and represents a functional measure for retinal ischemic damage as a result of the reduction of blood flow or blood pressure [3]. Accordingly, the oscillatory response has been suggested to represent a valid measure of the functional integrity of the microcirculation of the inner retina [4]. Moreover, pattern ERG (PERG) has the ability to quantify retinal ganglion cell function in the course of ischemia [5].

The clinical associations between retinal ischemia and internal carotid artery stenosis (ICAS) are well established. The ophthalmic manifestations of carotid insufficiency encompass a heterogeneous spectrum of clinical manifestations that varies according to the ICAS extent and its duration, including transient episodes of monocular blindness (referred to as amaurosis fugax), venous stasis retinopathy, ocular ischemic syndrome with rubeosis iridis, and the development of neovascular glaucoma [6]. The most common ocular symptom, which occurs in 30–40% of patients with atherosclerotic carotid occlusive disease, is ipsilateral transient visual loss [7]. More than 90% of patients with transient monocular visual loss have abnormal carotid angiograms [8]. Furthermore, affected patients are also at risk for developing ocular/orbital pain, aqueous flare, and cataracts. Fluorescein angiography has demonstrated delayed and patchy choroidal filling and diffuse leakage from the retinal vessels and the optic nerve head [9]. Interestingly, ocular signs and symptoms may represent the first manifestation of carotid stenosis [7].

It is worth noting that in general, the retinal examination of patients with ICAS is performed in direct time relation to the occurrence of clinical ocular symptoms. However, we hypothesize that prior to the development of the clinical ocular symptoms associated with ICAS, the choroid and retina may be affected by ischemia that results in subclinical electrophysiological retinal dysfunction. It may even represent its unique detectable manifestation. The incidence of abnormal retinal functioning in asymptomatic ICAS in the course of atherosclerotic carotid artery disease has previously been investigated using a full-field ERG and multifocal ERG (mfERG) to a limited extent [10, 11]. As ERG and mfERG originate from preganglionic elements [12], these electrophysiological tests do not enable the functional evaluation of the innermost retinal layers, particularly the macular region. In contrast, the function of ganglion cells and their fibers may be specifically assessed by pattern electroretinogram (PERG) and pattern visual evoked potentials (PVEP) [13]. Nevertheless, PERG and PVEP results in the eyes of patients with significant ICAS are currently unknown.

Therefore, the present study is designed to assess retinal and optic nerve bioelectrical function using ERG, combined with PERG and PVEP in both eyes of patients with unilateral asymptomatic but hemodynamically significant ICAS without objective signs and symptoms of ocular ischemia.

## 2. Material and Methods

### 2.1. Characteristics of the Study Group

Eighty-four eyes of 42 subjects with a diagnosis of unilateral hemodynamically significant ICAS (i.e., ≥70% of ICA diameter reduction in neurologically asymptomatic patients, defined as subjects, who lack chronic or transient ischemic symptoms typical for the carotid territory or transient ipsilateral blindness in the previous six months prior to study recruitment) were analyzed. The typical carotid territory ischemic symptoms included contralateral weakness of the face, arm, leg, or both and contralateral sensory deficit or paresthesia of the face, arm, leg, or both as a result of a developed stroke or transient ischemic attack (TIA) [14]. The subjects were randomly selected from a group of patients in the Vascular Surgery Department of Pomeranian Medical University in Szczecin, Poland who qualified for carotid endarterectomy (CEA). Thirty-four age- and sex-matched volunteers without signs of carotid stenosis or exceptionally with ICAS at a grade lower than 40% were enrolled as the control group. The degree of each individual's ICAS was diagnosed via carotid color Doppler ultrasonography (CDU) using a Voluson 730 PRO, GE Medical Systems device (Milwaukee, WI, USA), with a multifrequency 7.5-MHz linear probe, in accordance with the guidelines recommended in the literature [15, 16]. Patients with ocular ischemic syndrome or evidence of concomitant eye and chronic systemic disease that potentially affects the retina and optic nerve, that is, glaucoma, intraocular inflammatory diseases, retinopathy, recent (within 3 months) ocular surgery, advanced cataract, collagen-related or neoplastic disease, or a history of previous contralateral/unilateral CEA or ICAS stenting were excluded from the study. All enrolled subjects underwent a complete ophthalmic examination of both eyes, that is, distant best-corrected visual acuity (DBCVA), intraocular pressure (IOP) measurements, and anterior segment and dilated ophthalmoscopy fundus examinations using slit lamp biomicroscopy. The ophthalmological inclusion criteria included a best-corrected distance visual acuity (VA) > 0.8 (Snellen chart) and normal results with respect to a routine ophthalmological examination.

Data regarding medical history, current drug use, and smoking status were collected based on laboratory data, pathology tests, and other information, with particular focus on heart and vascular conditions and preexisting arterial hypertension. Furthermore, the actual arterial blood pressure (BP) was directly measured prior to ophthalmic examination in all subjects using a noninvasive blood pressure system with a manual aneroid manometer. The mean result from three measurements obtained with 5-minute-resting intervals was calculated. From the obtained BP data, the systemic mean arterial pressure (MAP) was calculated as follows: MAP = diastolic  BP + 1/3 (systolic BP − diastolic BP) mmHg. Furthermore, the following medical parameters were assessed in all patients: waist circumference [cm], waist/hip ratio (WHR), and body mass index (BMI) [weight (kg)/height (m)2]. Cumulative pack-years were calculated using the reported average number of cigarettes smoked per day and the number of years of smoking.

The study adhered to the tenets of the Declaration of Helsinki, and approval was obtained from the Local Research Ethics Committee. Moreover, each patient provided written informed consent for his or her involvement.

### 2.2. Electrophysiology

Full-field electroretinograms (UTAS-E 2000 system, LKC Technologies, Inc.), pattern electroretinograms, and pattern visual evoked potentials (RetiPort System, Roland Consult Instr.) were recorded according to the International Society for Clinical Electrophysiology of Vision (ISCEV) protocols [17–19]. For full-field ERG, recordings were performed binocularly with the use of bipolar Burian-Allen electrodes (Hansen Ophthalmic Development Lab, Iowa, USA) and a ground clip gold electrode (Natus Europe GmbH, Planegg, Germany) attached to the earlobe. Prior to recording, the cornea was anesthetized (Alcaine; Alcon) and the pupils were dilated with 1% tropicamide eye drops. The recording system parameters were as follows: amplifier sensitivity, 10–20–50 *μ*V/div; filters, 0.3–500 Hz (for OP extraction: 75–500 Hz); notch filters, off; time base, 5 ms/div; and artifact reject threshold, 0 *μ*V. A 30 min dark adaptation preceded the rod and mixed cone-rod recordings; a 10 min period of light adaptation preceded the photopic cone and flicker recordings. The rod ERG recordings were obtained with single dim white flashes of 1.6 cd s m^−2^ attenuated by a 24 dB neutral filter. The mixed rod-cone ERGs were elicited with flashes of white light of 1.6 cd s m^−2^. The cone ERGs were elicited with white flashes of the same intensity. In the flicker recordings, the frequency of stimulation was 30 Hz (the same conditions of adaptation) and ten sweeps were averaged. The oscillatory potentials in the scotopic state were obtained with 1.6 cd s m^−2^ white flashes. The overall wave index of the OP amplitudes (O1 + O2 + O3) was measured.

For PERG, monocular stimulation was used, in combination with an appropriate refractive error correction in relation to the eye-screen distance. The examination was interrupted when frequent blinking or fixation losses were identified (the patient was monitored with a TV camera). The pupils were not dilated, and central fixation was used. The PERG stimulation parameters were as follows: a 21″ CRT monitor with a frame rate equal to 70 fps was used; dimension of the stimulus field was 15024′ (the mean of the width and the height of the screen), with the aspect ratio between the width and the height (screen proportion H/V) equal to 4 : 3; black and white reversing checkerboard was presented to the patient, with a check size equal to 0048′; luminance for white elements was equal to 120 cd/m^2^, mean luminance of the stimulus screen: 62 cd/m^2^, with Michelson contrast set to 97%; temporal frequency was equal to 4.0 rps (2.0 Hz). The electrodes were as follows: a ground (gold disk) electrode (Roland Consult, Brandenburg, Germany) was placed on the forehead (Fpz), a thread Dawson-Trick-Litzkow (DTL) electrode (Diagnosys LLC, MA, USA) was used as active, and a gold disk placed at the ipsilateral outer canthus was used as a reference. The recording system parameters were as follows: amplifier sensitivity −20 *μ*V/div, filters: 1–100 Hz, artifact reject threshold −95% (for the amplifier range ±100 *μ*V), notch filters were off, averaging: 200 sweeps, and sweep time: 250 ms (time base: 25 ms/div). Two consecutive waveforms were recorded; they were subsequently averaged and analyzed off-line.

For pattern visual evoked potentials, monocular stimulation was used without pupil dilation and refraction correction was applied with respect to the eye-screen distance. Central fixation was used. Patient was monitored with a TV camera, and interruptions of the test were introduced when fixation loose or frequent blinking was observed. 21″ CRT monitor with a frame rate equal to 70 fps was used for pattern stimulation; dimension of the stimulus field was 170 (the height of the screen), with aspect ratio between the width and the height (screen proportion H/V) equal to 4 : 3; black and white reversing checkerboard was presented to the patient, with two check sizes equal to 0016′ and 104′; mean luminance of the stimulus screen was equal to 60 cd/m^2^, (luminance for white elements: ca. 120 cd/m^2^), with Michelson contrast set to 97%; temporal frequency was equal to 1.875 rps (0.938 Hz). The electrodes were as follows: an active-gold disk electrode (Roland Consult, Brandenburg, Germany) was placed on the scalp over the visual cortex at Oz with a reference-gold disk electrode placed at Fz; a ground (gold disk) electrode was placed on the forehead at fpz. Before recordings, interelectrode impedance was measured; values < 5 kΩ were accepted. Parameters of the recording channel were as follows: amplifiers range ±100 *μ*V/div, filter frequency bandwidth 1–100 Hz. Analysis period (sweep time) was equal to 300 ms. Artifact reject threshold was set to 95%, and 100 sweeps were averaged. For each eye and each check size, two consecutive PVEP waveforms were recorded and off-line averaged for further analysis.

### 2.3. Optical Coherence Tomography

The macular and peripapillary retinal nerve fiber layer (RNFL) thicknesses were measured before and 3 months after endarterectomy for each patient, using optical coherence tomography (Cirrus OCT, Zeiss Humphrey System, Dublin, California, USA). A macular profile of the central 6 mm zone was obtained using a fast macular scan protocol. The central subfield thickness (CST), cube average thickness (CAT), and cube volume (CV) automatically measured by a retinal border detection algorithm in the software were used for statistical analysis.

### 2.4. Statistical Analysis

Parameters were compared between independent groups with the Mann–Whitney test for quantitative variables and Fisher's exact test for qualitative variables. The Wilcoxon signed-rank test was used to compare parameters between the eyes ipsilateral and contralateral to ICAS. The strength of the association between the quantitative variables was measured with Spearman rank correlation coefficient (Rs). A *p* < 0.05 was considered statistically significant.

## 3. Results

Eighty-four eyes of 42 patients with significant unilateral ICAS were included. Thirty-four controls had both eyes analyzed. The eyes were organized into 3 groups: EIS—eyes ipsilateral to ICAS (*n* = 42), ECS—eyes contralateral to ICAS (*n* = 42), and controls without ICAS (*n* = 68). The mean extent of internal carotid stenosis was 79 ± 12%.

The clinical characteristics of the patients and controls are summarized in Table 1. The ICAS and control groups were matched for age, gender, and selected, well-known atherosclerotic risk factors, including hypertension, history of ischemic heart disease, cardiac infarction, peripheral artery disease, and aortic aneurysm. The rate of previous stroke was significantly increased in the ICAS group compared with the control group (33.33% versus 8.82%, resp.). Moreover, there was no significant difference in the values of the BMI, WHR, MAP, and proportion of active and former smokers between the groups.

Table 2 provides the values of the IOP and OCT parameters obtained for all investigated groups of eyes. The IOP values were significantly smaller in the EIS and ECS compared with the controls. In the macula, both the cube average thickness and cube volume values were significantly reduced both in the EIS and ECS compared with the controls. Importantly, no significant difference was identified between the eyes from the EIS and ECS groups in the patients with ICAS. Moreover, there was no difference in the value of peripapillary RNFL in the EIS and ECS groups compared with the controls.

Table 3 indicates the values of the PERG and PVEP measurements obtained in the eyes of all investigated groups. The amplitudes of the PERG P50 and N95 waves were significantly smaller in the EIS and ECS compared with the controls. No significant difference in both amplitudes was identified between the eyes from the EIS and ECS groups in the patients with ICAS. When analyzing the VER parameters, the amplitudes and peak times of the P100 waves did not significantly differ compared with the controls.

The values of the full-field ERG amplitudes and peak times for all groups are summarized in Table 4. The rod b-wave amplitudes were significantly decreased (*p* = 0.01), and the implicit times were significantly prolonged (*p* = 0.0004) in the EIS group compared with the controls. Similarly, in the ECS group, the values of the rod b-wave implicit times were significantly prolonged (*p* < 0.0001) compared with the controls. Furthermore, in the EIS and fellow eyes, we identified a significant decrease in the rod-cone a-wave amplitudes (*p* = 0.05 and *p* = 0.008, resp.) and a delay in the a-wave peak times (*p* = 0.02 and *p* = 0.007, resp.). When the OP wave index was analyzed, a significant decrease in the amplitudes was identified exclusively in the EIS (*p* = 0.02). Remarkably, there were no differences in the full-field ERG wave amplitudes and implicit times between the EIS and ECS.

Furthermore, to characterize the other risk factors that may influence the electrophysiological function of the retina and optic nerve, we investigated the association between full-field ERG amplitudes and peak times and several systemic risk factors and conditions. We determined that increasing the WHR was associated with longer rod b-wave implicit times (Rs = +0.33, *p* = 0.03 in EIS; Rs = +0.36, *p* = 0.02 in ECS; and Rs = +0.25, *p* = 0.04 in controls). Thus, the WHR positively correlated with the rod-cone a-wave peak times (Rs = +0.15, *p* = 0.33 in EIS; Rs = +0.22, *p* = 0.15 in ECS; and Rs = +0.36, *p* = 0.002 in controls) and the rod-cone b-wave peak times (Rs = +0.13, *p* = 0.40 in EIS; Rs = +0.19, *p* = 0.22 in ECS; and Rs = +0.24, *p* = 0.04 in controls). Interestingly, the rod b-wave and rod-cone b-wave amplitudes, as well as the magnitudes of the OP wave index, were dependent on the amount of cigarettes smoked. In the control group, an increase in the smoking pack-years was associated with a lower OP wave index (Rs = −0.33, *p* = 0.006) and lower rod b-wave (Rs = −0.36, *p* = 0.002) and rod-cone b-wave (Rs = −0.5, *p* < 0.0001) amplitudes. Similarly, the magnitudes of both the PERG P50 and N95 wave amplitudes were correlated with the amount of cigarettes smoked. An increase in the smoking pack-years was associated with lower PERG P50 wave amplitudes (Rs = 0.49, *p* < 0.0001) and N95 wave amplitudes (Rs = −0.33, *p* = 0.006).

## 4. Discussion

Ocular circulation is predominately controlled by autoregulative mechanisms, which maintain a stable retina and, to a large extent, the choroidal and optic nerve blood supply. In the case of carotid atherosclerosis and hemodynamically significant internal carotid artery stenosis, chronic cerebral hypotension may induce a downward shift in the cerebral and ocular vascular autoregulation. A major part of the oxygen and glucose consumed by the retina is delivered by the choroid [20]. The choroid has one of the highest blood flows in the body; however, it has no effective autoregulation. Consequently, the outer retina is more susceptible to hypoxia as a result of systemic ischemia [21]. It has been demonstrated that patients with ICAS have thinner choroids compared with age-matched healthy individuals [22]. Similarly, choroidal hypoperfusion as a result of ICAS resulted in multiple occlusions of the choriocapillaris and attenuated choroidal vessels [23]. In contrast, retinal circulation through the central retinal artery is highly dependent on the blood concentration of O_2_ and CO_2_ and the pH levels in the inner retina [24, 25]. Retinal flux may be increased in hypoxia by up to 336% [24], which is not possible in the choroid.

It has been demonstrated that full-field a- and b-wave ERG amplitudes are reduced in ocular ischemic syndrome compared with healthy eyes [9]. More recently, Kofoed et al. identified significant delays in all first-order summed implicit times and a clear trend towards reduced amplitudes in mfERG compared with healthy controls [26]. In the present study, we demonstrated that even in the absence of the clinical signs of ocular ischemic syndrome, significant ICAS may be associated with retinal function abnormalities. We identified significantly decreased amplitudes and prolonged peak times of scotopic b-waves and scotopic/photopic a-waves, as well as a reduced OP wave index in eyes ipsilateral to ICAS compared with controls. It is widely accepted that the a-wave is primarily generated from photoreceptors, and the b-wave is generated from bipolar cells [27]. Oscillatory potentials (OPs) are small rhythmic waves superimposed on the ascending b-wave of the ERG, and amacrine and bipolar cells are directly or indirectly involved in their generation [4]. Thus, our findings suggest that the rod-mediated response is mainly affected as a result of significant carotid stenosis. It is worth noting that the outer retina is at an increased risk of hypoxic injury in the dark compared with the light because oxygen consumption in photopic conditions represents only 60% of consumption in a scotopic environment [28, 29]. Increased oxygen consumption is related to an increase in retinal blood flow from 40% to 70% following the transition from light to dark [30]. Our full-field ERG results are in agreement with the findings of other groups [10].

Our findings indicated that ICAS may cause impairment in macular function, which was demonstrated by the significant reduction in the PERG P50 and N95 wave amplitudes. PERG has clinically been used to evaluate macular function because it is a focal response that reflects the activity of directly stimulated retinal areas and is thought to be correlated mainly with ganglion cell activity [31]. The objective assessment of macular function using PERG permits the practical differentiation between maculopathy and optic neuropathy in different clinical settings [32]. To the best of our knowledge, PERG testing has not been previously used to evaluate preganglionic macular function in patients with significant carotid stenosis. The previously published study performed with the use of mfERG in patients with unilateral carotid artery stenosis and without clinical eye disease demonstrated significantly reduced and delayed response in electroretinogram, which was correlated with an increasing grade of carotid artery stenosis [11]. Interestingly, cone function deviations have been demonstrated to correlate with actual arterial blood pressure [11]. These findings may suggest that the macula is affected well before the onset of clinical eye disease. Our results indicate the importance of PERG testing in the evaluation and management of patients with asymptomatic severe ICAS to identify macular dysfunction as a result of ICAS-related ischemic conditions.

Interestingly, impaired macular function was accompanied by decreased macular thickness and volume compared with the control group as detected in SD-OCT images in our study. Thus, we speculate that a reduction in PERG wave amplitudes is associated with a permanent loss of retinal cells in the macular region. Sepah et al. demonstrated a strong relationship between retinal function and the thicknesses of individual retinal layers based on the correlation between retinal parameters on OCT images and functional loss detected in ERG [33]. Interestingly, we identified reduced intraocular pressure in eyes ipsilateral and contralateral to ICAS compared with controls. This finding is in concordance with a previous report, in which carotid artery stenosis reduced the blood supply to the eye, which significantly affected several blood flow parameters, such as the intraocular pressure, ocular systolic pressure, and ocular perfusion pressure [11]. Remarkably, the magnitudes of both the ERG and PERG amplitudes were dependent on the amount of cigarettes smoked in our study. The effects of cigarette smoking on PERG amplitudes and implicit times in habitual smokers have been previously reported [34]. There are several mechanisms by which cigarette smoking negatively affects the retina: increased oxidative stress, lipid peroxidation, and platelet aggregation, as well as a reduction in plasma high-density lipoprotein or antioxidant levels [35, 36]. It is worth noting that WHR was negatively correlated with the rod b-wave implicit times of full-field ERG in our study. This finding indicates that obese patients had longer peak times of the rod-mediated response compared with their lean counterparts. Indeed, the retinal function of patients with hyperlipidemia was significantly decreased compared with healthy controls, even before the occurrence of pathological changes in the fundus, and BMI values were negatively correlated with the OP wave index [37].

Carotid arteries are the main blood vessels that deliver blood to the brain and the remaining head organs; blockage in the carotid artery decreases blood flow to the brain and the orbits. It has previously been reported that in cases of severe unilateral ICAS, the orbital and ocular blood flow velocities, visual functional, and morphological parameters have symmetrical patterns in both eyes and are generally reduced [38, 39]. This finding has been attributed to flow redistribution via the circle of Willis, which supplies several collateral pathways that determine the balance of flow between the two cerebral hemispheres and between the anterior and posterior cerebral circulations [40]. The presence of collateral circulatory pathways is crucial to maintain the cerebral perfusion pressure, metabolism, and function. The external carotid artery via reversed flow in the ophthalmic artery and leptomeningeal anastomoses further contribute to the collateral circulation [40]. This finding is in concordance with our observations in which no differences were identified in the PERG, PVER, or full-field ERG wave amplitudes or implicit times between the EIS and fellow eyes of patients with unilateral significant ICAS. Similarly, there were no discrepancies in the retinal morphology in the OCT images and IOP between both eyes of the same patient with severe ICAS. These findings may indicate that a reduction in the blood inflow to the head as a result of unilateral ICAS affects the hemodynamic blood flow conditions both in the ipsilateral and fellow eyes.

Reliable measurement of retinal function is needed to provide information beyond that which could be obtained by routine ophthalmological examination. It has been proved that ERG responses ensure a good function of whole retina, and the mfERG assesses the macular function. Recently, visual electrophysiological tests were proposed to evaluate preoperative macular function, which is used to predict the outcome of cataract surgery [41] or as a viable indicator of drug penetration into the central nervous system through the blood-neural barriers [42]. Our findings would support the notion of using the ERG testing also as a new biomarker to assess retinal blood flow conditions that can be affected by hemodynamically significant ICAS in apparently healthy eyes. Thus, preoperative visual electrophysiological tests would be essential for determining whether and when the carotid surgery is necessary for asymptomatic ICAS.

A methodological limitation of our study design was the analysis of both eyes for each control to achieve maximal statistical power for comparison with EIS and ECS eyes. Due to the fact that parameters of electrophysiological function for the right and left eyes of the same control were not independent, such procedure might overestimate statistical significance of differences between ICAS and non-ICAS eyes. Unfortunately, we could not use relevant parametric tests to adjust for the correlations between both eyes of each subject since, as noted above, distributions of the parameters deviated from normal distribution. The procedure where each eye is treated as a separate item for the statistical analysis is widely used in ophthalmology in spite of the methodological objections.

## 5. Conclusions

Our study demonstrated that retinal bioelectrical function is negatively affected by hemodynamically significant ICAS despite the absence of the objective clinical signs and symptoms of ocular ischemia. ERG and PERG testing appear to be valuable tools for the detection of retinal ischemia as a result of severe carotid stenosis and may represent a clinically useful adjunct to standard algorithms in the management of asymptomatic ICAS patients, particularly in the consideration of carotid surgery.

## Figures and Tables

**Table 1:** Clinical characteristics of the study groups.

Parameter	ICAS group	Control group	*p* value^a^
Number of subjects	42	34	—
Sex (male/female)	28/14	21/13	0.81
Patient's age [years] (mean ± SD)	62.5 ± 6.5	62.5 ± 4.0	0.80
BMI [kg/m^2^] (mean ± SD)	27.91 ± 3.67	27.34 ± 3.63	0.30
WHR [arbitrary units] (mean ± SD)	0.94 ± 0.11	0.93 ± 0.11	0.52
Waist circumference [cm] (mean ± SD)	97.24 ± 13.31	97.47 ± 13.24	0.98
MAP [mmHg] (mean ± SD)	104.30 ± 9.87	104.05 ± 11.07	0.96
Current smokers (%)	33.33	23.53	0.45
Former smokers (%)	85.71	76.47	0.38
Smoking pack-years (mean ± SD)	31.05 ± 30.94	27.18 ± 24.48	0.67
Period without smoking [years] (mean ± SD)	5.36 ± 6.86	7.40 ± 10.59	0.99
Hypertension (%)	80.95	64.71	0.12
Duration of hypertension [years] (mean ± SD)	5.66 ± 6.06	7.68 ± 8.51	0.75
History of ischemic heart disease (%)	35.71	20.59	0.20
History of cardiac infarction (%)	16.67	17.65	1.00
History of cerebral stroke (%)	33.33	8.82	**0.01**
History of peripheral artery disease (%)	23.81	23.53	1.00
History of aortic aneurysm (%)	9.52	11.76	1.00

^a^Mann–Whitney test for quantitative variables and Fisher's exact test for qualitative ones.

**Table 2:** Mean values (±standard deviation) of IOP and OCT parameters in EIS, ECS, and control eyes.

Parameter	EIS *n* = 42	ECS *n* = 42	Control *n* = 68	*p* value		
				EIS versus control^a^	ECS versus control^a^	EIS versus ECS^b^
IOP (mmHg)	15.27 ± 2.65	15.48 ± 2.36	16.57 ± 2.49	**0.006**	**0.03**	0.70
CST (*μ*m)	268.69 ± 23.0	265.293 ± 21.7	269.9 ± 20.1	0.87	0.39	0.62
CV (mm^3^)	9.90 ± 0.45	9.89 ± 0.52	10.06 ± 0.43	**0.04**	**0.03**	0.64
CAT (*μ*m)	276.95 ± 12.72	276.78 ± 14.42	281.81 ± 11.99	**0.03**	**0.03**	0.60
RNFL thickness (*μ*m)	91.50 ± 8.30	92.10 ± 10.46	93.72 ± 8.78	0.21	0.27	0.50

^a^Mann–Whitney test; ^b^Wilcoxon signed-rank test.

**Table 3:** Mean values (±standard deviation) of PERG and PVEP amplitudes and peak times in EIS, ECS, and control eyes.

Parameter	EIS *n* = 42	ECS *n* = 42	Control *n* = 68	*p* value		
				EIS versus control^a^	ECS versus control^a^	EIS versus ECS^b^
PERG						
N35-P50 amplitude (*μ*V)	2.78 ± 1.67	2.63 ± 2.02	3.65 ± 1.51	**0.01**	**0.0002**	0.19
P50 peak time (ms)	53.87 ± 4.95	52.64 ± 4.13	54.19 ± 3.16	0.47	0.03	0.57
P50-N95 amplitude (*μ*V)	4.22 ± 2.97	3.75 ± 2.91	5.62 ± 2.24	**0.002**	**<0.0001**	0.06
PVEP						
P100 amplitude (*μ*V) (1°4′)	7.1 ± 3.93	7.21 ± 3.97	7.96 ± 3.47	0.13	0.20	0.58
P100 amplitude (*μ*V) (0°8′)	8.63 ± 5.75	8.4 ± 4.8	9.15 ± 5.13	0.43	0.43	0.34
P100 peak time (ms) (1°4′)	112.91 ± 10.84	114.5 ± 8.63	111.23 ± 8.48	0.52	0.07	0.13
P100 peak time (ms) (0°8′)	118.24 ± 11.95	119.57 ± 11.42	118.83 ± 8.2	0.63	0.44	0.65

^a^Mann–Whitney test; ^b^Wilcoxon signed-rank test.

**Table 4:** Mean values (±standard deviation) of full-field ERG amplitudes and peak times in EIS, ECS, and control eyes.

Parameter	EIS *n* = 42	ECS *n* = 42	Control *n* = 68	*p* value		
				EIS versus control^a^	ECS versus control^a^	EIS versus ECS^b^
Amplitude (*μ*V)						
Rod b-wave	82.08 ± 38.01	92.02 ± 38.99	98.6 ± 27.84	**0.01**	0.096	0.09
Rod-cone a-wave	159.46 ± 57.3	148.82 ± 49.18	177.15 ± 48.38	**0.05**	**0.008**	0.17
Rod-cone b-wave	381.18 ± 84.5	380.04 ± 88.6	397.09 ± 67.48	0.26	0.14	0.84
OPs wave index	98.05 ± 28.95	100.24 ± 28.25	114.46 ± 34.86	**0.02**	0.08	0.40
Cone a-wave single flash	26.56 ± 11.55	24.51 ± 11.81	29.44 ± 8.03	0.12	**0.01**	0.49
Cone b-wave single flash	83.87 ± 29.17	82.06 ± 25.93	89.18 ± 22.88	0.23	0.09	0.85
Cone b-wave 30 Hz flicker	70.07 ± 26.85	66.39 ± 26.31	76.64 ± 18.63	0.07	**0.008**	0.13
Peak time (ms)						
Rod b-wave	112.12 ± 10.73	113.99 ± 9.65	105.71 ± 8.03	**0.0004**	**<0.0001**	0.12
Rod-cone a-wave	24.31 ± 1.58	24.32 ± 1.6	23.9 ± 1.07	**0.02**	**0.007**	0.91
Rod-cone b-wave	48.1 ± 3.45	48.11 ± 3.84	47.68 ± 2.83	0.11	0.09	0.85
Cone a-wave single flash	16.13 ± 1.51	16.11 ± 1.59	15.95 ± 1.2	0.74	0.87	0.96
Cone b-wave single flash	32.2 ± 1.92	32.23 ± 1.99	32.03 ± 1.64	0.68	0.43	0.77
Cone b-wave 30 Hz flicker	33 ± 0.86	33.05 ± 0.79	32.89 ± 1.06	0.82	0.89	0.99

^a^Mann–Whitney test; ^b^Wilcoxon signed-rank test.
